# A corpus-based study on Chinese and American students' rhetorical moves and stance features in dissertation abstracts

**DOI:** 10.3389/fpsyg.2022.1004744

**Published:** 2022-11-04

**Authors:** Yingliang Liu, Xuechen Hu, Jiaying Liu

**Affiliations:** School of Foreign Languages, Wuhan University of Technology, Wuhan, China

**Keywords:** stance markers, rhetorical moves, dissertation abstracts, Chinese doctoral students, American doctoral students

## Abstract

Dissertation is the most important research genre for graduate students as they step into the academic community. The abstract found at the beginning of the dissertation is an essential part of the dissertation, serving to “sell” the study and impress the readers. Learning to compose a well-organized abstract to promote one's research is therefore an important skill for novice writers when they step into the academic community in their discipline. By comparing 112 dissertation abstracts in material science by Chinese and American doctoral students, this study attempts to analyze not only the rhetorical moves of dissertation abstracts but also the lexical-grammatical features of stance in different abstract moves. The findings show that most of the abstracts include five moves, namely, Situating the research, Presenting the research, Describing the methodology, Summarizing the findings, and Discussing the research. However, fewer abstracts by Chinese students include all five moves. In addition, the choices of stance expressions by the two groups vary across the five abstract moves for different communication purposes. The results of this study have pedagogical implications for facilitating the development of academic writing skills for L2 writers.

## Introduction

Theses and dissertations are the most important research genres for master's and Ph.D. students as they step into the academic community. Swales (2004, p. 99) commented that “the dissertation/thesis is the most sustained and complex piece of academic writing (in any language) they will undertake,” especially for non-native English speakers (Sükan and Mohammadzadeh, 2022). As an abstract is a highly condensed and conventionally structured text, the knowledge of the rhetorical moves of abstracts and linguistic sources to realize these moves is essential for novice writers, particularly for L2 writers (Pho, 2008).

Swales (1990) defines the abstract as both a summary and a “purified” reflection of the entire article. In addition to the informative function, abstracts also function to promote the research to the readers. Therefore, it is important to understand the reader-writer interaction and the linguistic realization of the promotional function in abstracts. Much attention has been paid to the rhetorical moves (Santos, 1996; Can et al., 2016; Tankó, 2017; Amnuai, 2019) and linguistic characteristics of abstracts in research articles (Ebrahimi and Chan, 2015; Friginal and Mastafa, 2017; Ansarifar et al., 2018). Dissertation abstracts, however, differ significantly from research article abstracts in the subcategories of moves and use of metadiscourse features (El-Dakhs, 2018), and little is known about how L2 graduate writers employ linguistic features to engage readers in dissertation abstracts. The present study, therefore, aims at exploring not only the rhetorical moves of dissertation abstracts by L2 graduate students but also the lexical-grammatical features of stance in different abstract moves.

## Literature review

### Move structure of abstracts

The significance of the abstract in academic writing lies in the variety of functions and roles it plays (Ebrahimi and Chan, 2015; Friginal and Mastafa, 2017; Nasseri and Thompson, 2021; Sükan and Mohammadzadeh, 2022). Firstly, it saves the reader's time by providing a condensed version of the whole research article (RA). Secondly, it helps the readers to decide on whether to continue to read the article. Thirdly, it has a persuasive function in convincing journal editors to accept a research article. Lastly, it also aids in indexing, which assists the research article to be easily located in the database. Dissertation abstracts can hold a higher stake than RA abstracts as the direct reader of dissertations is the reviewer who evaluates the quality of research and writing.

Following Swales's (1990) move analysis, a five-move pattern of abstracts was first proposed by Santos (1996) which includes Situating the research (M1), Presenting the research (M2), Describing the methodology (M3), Summarizing the results (M4), and Discussing the research (M5). Hyland (2000), based on an analysis of 800 RA abstracts from eight disciplines, proposed a similar five-move model: introduction, purpose, method, product, and conclusion (IPMPC). In the same line of research, Lorés (2004) found that 61% of 36 abstracts in the field of applied linguistics follow the IMRD structure (introduction-method-result-discussion), while 31% of them employ the CARS model (creating a research space), and 8% show a mixture of the two structures.

It has been attested that Ph.D. thesis abstracts and RA abstracts are two distinct genres, with the former representing an educational genre, and the latter, a professional one (Kawase, 2015; El-Dakhs, 2018). El-Dakhs (2018) compared Ph.D. thesis abstracts and RA abstracts in applied linguistics and found that thesis abstracts included lengthier introductions while RA abstracts provided a lengthier description of the methodology, findings, and implications. Afzaal et al. (2019) noted that EFL learners in Pakistan mostly followed Swales' CARS model in the abstract of master's dissertations, but they used past participle in the move of occupying the niche, suggesting a lack of confidence when presenting their own study. A review of these studies suggests that it is insufficient for novice writers to understand the generic move structure of abstracts. It is equally important for them to know how to realize rhetorical functions through linguistic devices.

### Stance in academic writing

Stance, a common feature concerned with writers' or speakers' personal attitudes, feelings, or judgments about the information communicated, has received increasing attention in recent years (Hyland, 2005b; Biber, 2006; Du Bois, 2007; Aull, 2019; Kaltenböck et al., 2020; Zhang and Zhang, 2021). Stance markers refer to the way that stance is expressed or realized, including a range of grammatical devices like modal auxiliaries, adverbial hedges, and complement clauses (Poole et al., 2019). They function to express different feelings such as the attitudes that a speaker or writer has about the information (Biber, 2006). Stance and stance markers play significant roles in academic writing but pose a challenge to academic writers. The author not only needs to guarantee the credibility of the research and make objective comments while reporting results or findings but also has to establish an authorial presence to interact with readers as well as acquire visibility in this field (Can and Cangir, 2019).

The past decade has witnessed a growing interest in stance and stance markers in academic writing. Following this line of research are investigations of a variety of linguistic devices, such as boosters and hedges (Hyland, 2012), personal pronouns (Yang, 2016; Can and Cangir, 2019), reporting verbs (Hyland, 1999, 2002; Peng, 2019), and engagement sources (Lancaster, 2014). The results have confirmed that academic writing, despite its expected objectivity, involves the expression of an authorial stance, including evaluation of previous studies, commitment to one's own claim, as well as attitudes toward one's own research.

The identification of stance features is highly context-dependent, which can lead to fuzziness in the classification. Biber (2006), therefore, examines stance from a lexical-grammatical approach which focuses on three categories of evaluative language: modal and semi-modal verbs, stance adverbs, and complement clauses controlled by stance verbs, adjectives, or nouns. This classification is more comprehensive and specific in identifying stance features and has been adopted in several studies on stance in academic writing (Chan, 2015; Çakir, 2016).

Stance in academic writing has been mainly examined from two perspectives: cross-disciplinary (Hyland, 2005b; Chan, 2015; Yang, 2016; Li, 2020) and cross-linguistic (Hu and Cao, 2011; Mur-Dueñas, 2011; Kim and Lim, 2013; Jiang, 2015; Loi et al., 2016; Bax et al., 2019). A comparison of research articles in different disciplines has shown that soft disciplines generally employ more stance markers than hard disciplines (Hyland, 2005b; Chan, 2015). Interestingly, a diachronic study of stance has found that the frequencies of stance markers are increasing in the sciences and falling in the soft disciplines, demonstrating a converging tendency (Hyland and Jiang, 2016).

As stance markers are used by writers to “engage with the socially determined positions of others” (Hyland, 2005a, p. 52), the deployment of these linguistic sources is expected to vary across different linguistic and cultural communities. For instance, Chinese journal articles are found to include fewer metadiscourse features, especially interactional metadiscourse features (hedges, boosters) than English research articles (Mu et al., 2015). The lower density of metadiscourse features in Chinese journal articles is also consistent in different parts of the articles, including abstracts (Hu and Cao, 2011) and introductions (Kim and Lim, 2013). These findings suggest the avoidance of authorial presence in a reader-responsible context.

In such a context, Chinese ESL (English as a second language) learners may transfer these norms from Chinese academic writing to English academic writing. As Hyland (2004, p. 141) argues, “the ways that writers present themselves, negotiate an argument, and engage with their readers are closely linked to the norms and expectations of particular cultural and professional communities.” Many existing studies have revealed difficulties in expressing the stance encountered by Chinese ESL students (Li and Warton, 2012; Lee and Deakin, 2016; Afzaal et al., 2021). Unlike their L1 peers, Chinese ESL students are found to maintain a more detached and impersonal writing style, and they are less strategic in making assertions (Lee and Deakin, 2016; Yoon, 2020). Most of these findings are based on argumentative writing by L2 students. As abstracts are persuasive in nature, it can be assumed that L2 novice writers may face similar challenges when drafting abstracts.

As mentioned in the last section, Ph.D. thesis abstracts differ from RA abstracts in the rhetorical structure. The linguistic realization of the rhetorical moves and the use of stance markers have also been found to be different. For example, more hedges and attitude verbs are found in Ph.D. thesis abstracts while the abstracts of journal articles include more impersonal markers and self-mentions (El-Dakhs, 2018). This difference suggests that compared to RA authors, who are in a higher power status in the scientific community, thesis writers as new members are more cautious in taking responsibility for their claims.

Although much attention has been paid to stance markers in academic writing and moves in abstracts, little is known about how novice L2 writers employ stance markers in the rhetorical moves in abstracts. In addition, most of the studies on abstracts have involved cross-linguistic or cross-disciplinary comparisons, and they have addressed disciplines in soft sciences such as applied linguistics (Hu and Cao, 2011; Can et al., 2016; Ansarifar et al., 2018; El-Dakhs, 2018; Nasseri and Thompson, 2021), literature, translation studies (Li, 2020), economics (Ebrahimi and Chan, 2015), and accounting (Amnuai, 2019). In contrast, abstracts in the hard sciences have received little attention. In universities in China, except for language-related studies, dissertations in other disciplines are written in Chinese. Nevertheless, abstracts in both English and Chinese are required. The dissertation abstract, therefore, is an ideal genre to examine EFL students' use of stance markers in academic writing. The purpose of the present study is to examine the use of stance markers in the moves structures of English abstracts written by Chinese and American doctoral students. Specifically, the present paper aims to answer the following two questions:

(1) Are there any similarities and differences between the features of move structure in Chinese and American doctoral students' dissertation abstracts?
(2) What are the lexical-grammatical features of stance in the five moves of Chinese and American students' dissertation abstracts?

## Methodology

### Data collection

The corpus-based study aims to investigate the lexical-grammatical features of stance in the move structures of English dissertation abstracts written by Chinese and American Ph.D. students. The data for this study include 112 English dissertation abstracts written by Chinese and American doctoral students majoring in materials science and engineering between 2010 and 2016. Two corpora were constructed as the corpus of Chinese students' abstracts (CCSA) and the corpus of American students' abstracts (CASA). CCSA included 56 dissertation abstracts extracted from the China Dissertation Database of Full Text. The dissertations were selected from universities in seven areas of China (i.e., North China, Central China, and Northwest China). The 56 dissertation abstracts of CASA were selected from the ProQuest Dissertation and Theses Database of Full text. The dissertations were selected from universities in four areas of America (i.e., North, South, West, and Midwest). Table 1 shows the overall information of the two corpora.

**Table 1:** Overall information of CCSA and CASA.

**Corpus**	**Number of abstracts**	**Word count**	**Average length**
CCSA	56	47,836	854
CASA	56	25,434	454

### Data analysis

All dissertation abstracts were analyzed in two ways. Firstly, Santos (1996) five-move structure model was employed to analyze the moves in the dissertation abstracts. The framework for the move structure is presented in Table 2 with one Chinese student's dissertation abstract used as an example. Ten abstracts were randomly selected and coded by the first and third authors separately. The coding was compared, and the agreement rate reached 98%. The application of the coding scheme was discussed. The third author then coded the rest of the data twice in 2 weeks intervals. All uncertain or inconsistent cases were discussed and settled among the three authors.

**Table 2:** Santos (1996) framework for move structure.

**Moves**	**Function/description**	**Example (CCSA-01)**
Move 1: Situating the research < STR >	Setting the scene for the current research (topic generalization)	With a stable six-membered ring structure, carbon nanotubes (MWNTs) have good thermal stability and play a flame retardant synergistic effect during the combustion of polymers.
Move 2: Presenting the research < PTR >	Stating the purpose of the study, research questions and/or hypotheses	On this basis, this paper presented carbon nanotubes was wrapped with silica microspheres, modified phosphazene, and ionic polymer respectively, by surface chemical modification. It studied the effect of MWNTs on the thermal stability and flame retardant of polymer and the flame-retardant mechanism during the combustion.
Move 3: Describing the methodology < DTM >	Describing the materials, subjects, variables, procedures	First part, two kinds of triazines charring agent (CA-ODA and CA-DDS) with high benzene ring content were synthesized by amino-substituted reaction.
Move 4: Summarizing the findings < STF >	Reporting the main finding of the study	With 4% of PAAZn-g-MWNTs, the PHRR of PMMA composites was reduced from 590 W·g^−1^ down to 355 W·g^−1^, and the total heat release rate was reduced from 25.5 kJ·g^−1^ down to 16.1 kJ·g^−1^.
Move 5: Discussing the research < DTR >	Interpreting the results/findings and/or giving recommendations, implications of study	It presented there was a synergistic effect between the MWNTs and modified phosphazene.

Secondly, stance markers in the dissertation abstracts were identified based on Biber (2006) classification of linguistic and grammatical features of stance, including modal and semi-modal verbs, stance adverbs, and stance-complement clauses controlled by verbs, adjectives, or nouns. AntConc software was used to identify the stance markers, and all identified instances were manually checked and classified into different sub-categories according to the semantic meaning revealed by the controlling stance word. Frequencies of the stance features were then normalized to a relative frequency per 10,000 words to account for the different sizes of the two corpora. The distribution of stance features in each abstract move was compared.

## Findings

### Move structure of abstracts in CCSA and CASA

To answer the first research question, moves in each dissertation abstract in CCSA and CASA were identified based on Santos (1996) five-move structure model. We first counted the number of moves in the abstracts to see if the abstracts included all five moves. The general move structure of abstracts in the two corpora is presented in Table 3.

**Table 3:** General move structure of abstracts in CCSA and CASA.

**Corpus**	**3 moves**	**4 moves**	**5 moves**	**Total**
CCSA	6 (10.71%)	16 (28.57%)	34 (60.71%)	56
CASA	3 (5.36%)	17 (30.36%)	36 (64.28%)	56

As seen in Table 3, most abstracts in both corpora include four or five moves (89.28% for CCSA and 94.64% for CASA). Very few abstracts have an incomplete structure with three moves. Fewer abstracts in CCSA include four or five moves than in CASA, though the average length of abstracts in CCSA is near twice the length of abstracts in CASA (see Table 1).

To examine the distribution of the five moves in the two corpora, the length and occurrence of each move were counted (see Table 4). As seen in Table 4, the largest portion of the abstracts in both corpora is devoted to the Summarizing the findings move, and the second and third largest portions are Describing the method and Situating the research respectively. Compared to American students, Chinese students put more emphasis on the research result with more than half of the abstract summarizing the findings, leaving very little room for discussing the research. In contrast, the length of each move in American students' abstracts is more balanced. On the other hand, the occurrence of each move is similar in both corpora. Most abstracts in both corpora include three obligatory moves: Presenting the research, Describing the methodology, and Summarizing the findings.

**Table 4:** Length and occurrence of each move in abstracts in CCSA and CASA.

**Moves**	**Average length of the move in CCSA**	**Average length of the move in CASA**	**Number of abstracts in CCSA containing the move (*n* = 56)**	**Number of abstracts in CASA containing the move (*n* = 56)**
Move 1: STR	15.18%	21.68%	53 (94.64%)	48 (85.71%)
Move 2: PTR	6.33%	7.25%	47 (83.93%)	55 (98.21%)
Move 3: DTM	20.28%	27.56%	56 (100.00%)	55 (98.21%)
Move 4: STF	53.49%	35.08%	56 (100.00%)	51 (91.07%)
Move 5: DTR	4.72%	10.39%	40 (71.43%)	48 (85.71%)

(The weight of the move in abstracts indicates the portion of the move out of the total word count of the abstract).

### Stance features of abstracts in CCSA and CASA

To answer the second research question, stance markers in both corpora were identified and classified, and then the normalized frequencies were calculated and compared. Biber (2006) divides stance markers into three categories: modals and semi-modals, stance adverbs, and stance-complement clause constructions. Table 5 presents the frequencies of the three major categories of stance markers in CCSA and CASA.

**Table 5:** Overall frequencies (per 10,000 words) of stance markers in CCSA and CASA.

**Stance markers**	**CCSA**	**CASA**	***X*** **-square**	***p*** **-value**
Modal and semi-modal verbs	59.58	39.32	13.04	0.00
Stance adverbs	21.53	31.45	6.56	0.01
Stance-complement clauses	68.57	81.39	3.77	0.05
Total	149.68	152.16	0.07	0.79

Table 5 shows no significant differences in the total number of stance markers between the two corpora (*p* > 0.05). Both groups use stance-complement clauses the most, and the American students employ slightly more of them than Chinese students (*p* = 0.05). The second preferred category for both groups is modal and semi-modal verbs and Chinese students employ significantly more of them than American students (*p* < 0.05). The least preferred category by the two groups is stance adverbs. The frequency of stance adverbs used by American students is higher than that of Chinese students.

As the most common category in both corpora, stance-complement clauses are further divided into verb-complement clauses, adjective-complement clauses, and noun-complement clauses. The results are presented in Table 6. A closer look at the sub-categories of the stance construction reveals some noticeable discrepancies between the two corpora. As shown in Table 6, both groups rely heavily on stance verb-complement clauses to show stance in the abstracts. Chinese students show a remarkable preference for the stance verb + *that*-clauses while American students use more stance verb + *to*-clauses than Chinese students. Stance-complement clauses controlled by adjectives and nouns were rarely found in the two corpora, although American students employed significantly more stance noun + *to*-clauses than Chinese students (*p* < 0.05).

**Table 6:** Normalized frequencies (per 10,000 words) of each class of stance-complement clauses in CCSA and CASA.

	**CCSA**	**CASA**	***X*** **-square**	***p*** **-value**
Stance verb + *that*-clause	50.59	34.99	8.982	0.00
Stance verb + *to*-clause	10.03	34.60	54.085	0.00
Stance adjective + *that*-clause	1.05	0.79	0.117	0.73
Stance adjective + *to*-clause	6.06	7.86	0.806	0.37
Stance noun + *that*-clause	0.42	1.18	1.411	0.24
Stance noun + *to*-clause	0.42	1.97	4.164	0.04

As for the choice of stance verb-complement construction in CCSA and CASA, it was noted that American students employed a similar amount of *that*- and *to*-clauses, whereas Chinese students preferred *that*-clauses to *to*-clauses. Significant differences were found between the two student groups in their use of stance verb + *that*-complement clauses and stance verb + *to*-complement clauses (*p* < 0.05). Specifically, in contrast to their American counterparts, Chinese students tend to overuse *that*-clauses controlled by communication verbs and epistemic certainty verbs (*p* < 0.05), but underuse *to*-clauses controlled by mental verbs and communication verbs (*p* < 0.05). In-depth analysis reveals that the stance verb + *that*-complement construction in both corpora is particularly common with communication verbs (e.g., *show, indicate, reveal*, and *demonstrate*) and verbs expressing epistemic certainty (e.g., *find, determine*, and *conclude*).

Example 1: The results ***show*** that antimony, tin oxide, ferric oxide can improve fire-resistance time and reduce smoke density (CCSA-02).

Example 2: Lastly, it was ***determined*** that the fabrication process greatly influences the impurity types and concentrations of the alloys, and therefore greatly dictate which thermal stability mechanisms care active (CASA-21).

In examples 1 and 2, stance verb + *that*-complement clauses are accompanied by inanimate subjects in both sentences. This structure is used to report information in an impersonal way, which conforms to the typical characteristics of academic writing. Both groups prefer *that*-clauses controlled by certainty verbs and communication verbs, which implies their confidence in the reported information.

Different from the use of stance verb + *that*-clauses in both corpora, the stance verb + *to*-complement construction is frequently employed with mental and communication verbs, especially among CASA.

Example 3: The Sb composition ***is found to*** vary non-linearly with substrate temperature and V/III ratios (CCSA-28).

Example 4: Instead, color center formation ***was shown to*** be controlled by oxidation and reduction of variable valence impurity ions, primarily Fe 2+/3+ (CASA-26).

As seen in examples 3 and 4, similar to *that*-clauses, stance verb + *to*-complement clauses usually appear with impersonal subjects, which demonstrates the objectivity and impartiality of the writers in declaring information. Nevertheless, the use of the mental verb *find* and communication verb *show* by the writers indicate a higher degree of certainty to the reported information, thus increasing the force of their claim. The two words *find* and *show* take up more than half of the communication and mental verbs in both corpora, indicating the students' inclination to employ these *to*-complement clauses to ascertain their confidence and certainty with regard to the information.

### Stance features in different moves of abstracts in CCSA and CASA

This section reports various types of stance markers across five moves of abstracts by both student groups. As shown in Table 7, the overall use of stance markers across the five moves in CCSA and CASA corpora have similar distribution characteristics. There are more stance markers in STR, STF, and DTR moves than in PTR and DTM moves in both corpora. It is noteworthy that American students use more stance markers in STR, PTR, DTM, and STF moves of abstracts than Chinese students, except for the DTR move. The distribution of stance markers is closely related to the different functions of each move. Situating the research (STR) move serves to set the scene or present background information for the study, which states the significance of the present study. Summarizing the findings (STF) move, which reports the findings and discusses the research part, shows a detailed interpretation of the results. However, presenting the research (PTR) move gives the hypothesis, and describing the methodology (DTM) move describes the methods of the study, in both of which an objective tone is expected.

**Table 7:** Normalized frequencies of stance markers across five moves (per 10,000 words).

**Stance markers**		**Modals**	**Stance adverbs**	**Stance verb-complement clauses**	**Stance adjective-complement clauses**	**Stance noun-complement clauses**	**Total**
STR	CCSA	77.11	20.66	34.43	24.79	1.38	158.36
	CASA	89.73	35.89	49.85	17.95	7.98	201.40
PTR	CCSA	3.30	0	3.30	0	0	6.61
	CASA	5.43	5.43	16.28	10.85	0	37.98
DTM	CCSA	5.15	1.03	4.12	0	1.03	11.34
	CASA	15.69	8.56	8.56	7.13	2.85	42.80
STF	CCSA	71.91	31.26	87.93	5.47	0.78	197.36
	CASA	19.05	44.83	133.36	5.60	1.12	203.97
DTR	CCSA	172.72	31.00	155.00	8.86	0	367.58
	CASA	98.37	56.75	90.81	3.78	3.78	253.50

The following sections provide a detailed examination of the distribution of different types of stance markers in every move of abstracts in CCSA and CASA.

#### Stance features in the STR move

The situating the research (STR) move in the abstracts serves to set the scene for the research and establish the significance of the current study (Hyland, 2000). As shown in Table 7, the distributions of stance markers in this move in the CCSA and CASA abstracts are similar. No significant differences in the frequencies of the five categories were identified between the two corpora (*p* > 0.05). In presenting this move, both groups of students favor modal verbs and stance verb-complement clauses the most, and stance noun-complement clauses the least. Further analysis shows that both groups prefer possibility modals in modal verbs the most and the other sub-classes of stance markers are remarkably less common than possibility modals.

Both Chinese and American students use various stance devices in this move to emphasize the significance of their research topic, which is useful for setting the scene for the present research. Particularly remarkable is the frequent use of modal verbs in the STR move. The possibility modal *can* is used with inanimate subjects to show ability or logical possibility meaning, as can be seen in examples 5 and 6.

Example 5: However, the performance of lithium ion batteries ***can*** no longer satisfy the ever increasing demands for high energy density even though they almost reach the theoretical limit both in mass and volume. Therefore, the improvement of the research for lithium ion batteries is urgent (CCSA-31).

Example 6: For instance, the ability to create controllable nanostructures in a scalable manner ***can*** enable the wide use of nanotechnology (CASA-07).

As shown in examples 5 and 6, the possibility modal *can* is utilized with inanimate subjects by both Chinese and American students. Example 5 stated that lithium-ion batteries fail to meet the demands, thus providing a rationale for the current study. Example 6 suggested the necessity to develop a new function of nanotechnology. The choice of *can* shows the possibility of conducting research related to this topic from different perspectives.

In contrast, the necessity modals (*must* and *should*) are less frequently employed than the possibility modals in the STR move in CCSA and CASA. Both *must* and *should* express the necessity, which can boost the claim expressed in the sentence. The word *must* is commonly used to express any unavoidable requirement or obligation. When they are employed in this move, the personal obligation voiced by them indicates the writer's emphasis on the necessity of the present study. This can explain why necessity modals appear more frequently in this move than in the other four moves of abstracts. For instance:

Example 7: The most efficient mode of transportation of petroleum-based fuel is *via* pipelines, and due to the 300% increase in ethanol use in the U.S. in the past decade, a similar method of conveyance ***must*** be adopted for ethanol (CASA-29).

Example 8: Therefore, the segregation behavior of B ***should*** be confirmed and the recovery rate of Si ***should*** be improved during the alloy solidification refining process (CCSA-07).

As seen in examples 7 and 8, *must* and *should* demonstrate the writers' advocacy of the action introduced in the clause, as well as the writers' confidence in claiming authority in the research area.

#### Stance features in the PTR move

As shown in Table 7, presenting the research (PTR) move includes the least stance markers in the two corpora partly because of its function to present the objective or hypothesis of the research in academic papers. The occurrence of different kinds of stance markers employed by American students is significantly higher than by Chinese students, with no exception (*X*^2^ = 6.116, *p* < 0.05).

It is noteworthy that both corpora contain more *to*-clause controlled by verbs of effort (*try, seek*) in the PTR move compared to the other moves. We can see in examples 9 and 10, the writers' employment of the stance resources demonstrates their purposes of stating the attempt and endeavor of their research effort, which is a useful way to accomplish the goal of presenting the research objective of the PTR move. For instance:

Example 9: In this thesis, we ***try to*** introduce a new weak (soft) phase on the basis of traditional nanoglass, to replace the role of grain boundaries, construct a new kind of nanoglass composite (CCSA-38).

Example 10: This investigation ***seeks to*** determine if current superalloy casting methods can result in the formation of oxide bifilms... (CASA-34)

In addition, some instances of epistemic likelihood verbs + *that* clauses and stance adjectives + *to* clauses exist in the PTR move of CASA. For instance:

Example 11: ***It was hypothesized that*** atmospheric pressure plasma treatment ***could*** functionalize and/or remove peel ply remnants on the CFRP surfaces to improve adhesion (CASA-54).

Example 12: Although the exact form of y(V) is unclear, ***it is reasonable to*** assume an exponentially-decaying form of y(V), where at low temperatures γ is... (CASA-31)

In example 11, the writer's uncertainty about the claim is revealed by the use of an extraposed *that*-clause controlled by the epistemic likelihood verb (*hypothesize*) and the combination of the modal verb with tentative meaning (*could*) in *that*-clause, which serves to present the hypothesis and indicate the writer's discretion in making the assumption. In example 12, the use of extraposed *to*-clause controlled by an adjective (*reasonable*) conveys a positive evaluation of this assumption. In both examples, the American students make the assumptions more convincing with the use of stance expressions. Although stance devices in the PTR move are the fewest in both corpora, the American students use more of these devices than the Chinese students to strengthen their personal involvement in the presentation of the research objective.

#### Stance features in the DTM move

Few stance expressions are found in describing the methodology move in both corpora. This is because the major function of this move is to introduce the methods and procedures for conducting the study. In this move, CCSA contains significantly fewer stance markers than in CASA (*X*^2^ = 16.450, *p* = 0.000), and Chinese students employ significantly fewer modal verbs, stance adverbs as well as stance adjective + *to*-clauses than the American students (*p* < 0.05). The possibility modals are the most common stance markers used by both student groups. For instance:

Example 13: The objective of these experiments was to correlate observations of slip transfer with a geometric parameter m', which ***can*** be used to identify and predict crystallographic arrangements that are better suited for slip transfer (CASA-03).

Example 14: So we introduce the method that ***can*** remove or weaken undesired carrier localization (CCSA-28).

In examples 13 and 14, the use of the possibility modal *can* in the description of methodology shows the justification of the methods employed. The considerably higher use of modals, especially possibility modals, in the DTM move of CASA than that of CCSA (*X*^2^ = 4.500, *p* < 0.05) suggests that the American students' greater efforts in justifying the choice of research tools and methods in this move. Regarding stance adverbs in DTM move, style adverbs and epistemic likelihood adverbs are preferred by American students. For instance:

Example 15: ***Specifically***, tensile stress generation was evaluated using an in situ wafer curvature measurement technique, and correlated with the inclination of edge-type threading dislocations observed with transmission electron microscopy (TEM) (CASA-24).

Example 16: In particular, the use of nanosphere lithography to pattern thin films of aluminum prior to anodization is described, which allows for ***nearly*** arbitrary control of the pore size, interpore spacing and aspect ratio (CASA-07).

The style adverb *specifically* in example 15 indicates the writer's attempt to describe the research methods to the readers in detail. The likelihood adverb *nearly* in example 16 shows the writer's caution when introducing the effectiveness of the research procedure. Both examples show American students' objectiveness in presenting the methodology, which is enhanced by stance adverbs.

#### Stance features in the STF move

Summarizing the findings (STF) move takes up the largest portion of the abstracts in both corpora and it involves the frequent occurrence of stance expressions. Results indicate that the American students employ significantly more stance verb-complement clauses (*X*^2^ = 13.837, *p* = 0.000), whereas the Chinese students use significantly more modals than the American students (*X*^2^ = 31.922, *p* = 0.000). Stance verb-complement clauses have the highest frequency in STF move in both corpora. In contrast, stance noun-complement clauses have the lowest frequency in both corpora. Unlike American students, Chinese students show a preference for modal verbs over stance adverbs in this move.

A close examination of the subcategories of stance expressions in the STF move shows that the stance verb + *that*-clauses is the most preferred device used by Chinese and American students. To be specific, *that*-complement clauses occur mostly after communication verbs (such as *indicate* and *show*) and epistemic certainty verbs (such as *find* and *determine*) in both groups. Examples 17 and 18 are two instances of stance verb + *that*-clause controlled by communication verbs *(indicate* and *show)* in the STF move.

Example 17: ***The simulations indicate that*** the particle size dependent velocity of the sedimentation increases the rate at which the system coarsens (CASA–10).

Example 18: ***The results show that*** the wear resistance of WC/Co-Cr composite coating is better than H13 steel (CCSA-23).

The communication verbs *show* and *indicate* are the most preferred stance verbs used by both students in STF move. Textual analysis reveals that stance verb + *that*-clauses controlled by communication verbs occur most frequently with inanimate subjects, such as “simulations” and “results” in examples 17 and 18. This shows the writers' objectivity and certainty toward their results when reporting their findings. Both student groups report the findings with words with high certainty to establish their personal expertise, thus making the findings more convincing.

In addition to the stance verb + *that*-clause, the stance verb + *to* clause, especially the *to*-clause controlled by mental and communication verbs, is also frequently used by American students in the STF move. However, this stance device is rarely found in Chinese students' abstracts. Significant differences were found between their overall use of stance verb + *to*-clauses (*X*^2^ = 91.104, *p* = 0.000), as well as their use of *to*-clauses controlled by mental and communication verbs (*p* = 0.000 for both). *To*-clauses in CASA were frequently controlled by mental verbs (such as *find, determine*, and *observe*) and communication verbs (such as *show* and *confirm*). Textual analysis shows that they often occur with subjects concerning the research-related object or process to denote objectiveness in the presentation of the findings (such as *find* and *show*), and the verbs selected are often with high certainty. For instance:

Example 19: The dissolution behavior of disordered Fe50Pd50 ***was found to*** strongly relate to the crystallographic orientation of specific grains (CASA-41).

Example 20: Finally, non-planar LEDs ***were shown to*** exhibit broader light-emission spectra than conventional planar LEDs (CASA-09).

In examples 19 and 20, the stance verb + *to*-clause in the STF move shares a similar role with *that*-clause, but the Chinese students seem to ignore this function of *to*-clauses in the STF move and tend to overuse *that*-clauses.

When presenting the findings of the current research, both Chinese and American students were prone to display their objectiveness and discretion to make the findings more reliable. Nevertheless, they also employ some constructions with high certainty to make the result appear more powerful and convincing. The Chinese students prefer verb + *that*-clauses to verb + *to*-clauses in STF move. In contrast, American students employ more stance verb-complement clauses with high certainty to make the research findings more powerful and forceful.

#### Stance features in the DTR move

Consistent with the argumentative nature, discussing the research (DTR) move in both corpora includes the most stance expressions. Results demonstrate that the two corpora are similar in the distribution of stance expressions, with modal verbs being the most frequently used and followed by stance verb-complement clauses and stance adverbs. Nevertheless, significant differences were found between the two corpora in the frequency of stance devices employed in this move. Specifically, the DTR move of CCSA contains significantly more modal verbs and stance verb-complement clauses (*p* < 0.05).

Modal verbs are the most preferred grammatical stance device in this move in both corpora, which are more frequent than in the other four moves.

Example 21: It proves that the wet-mix shotcrete with high air content ***can*** solve the inharmonious contradiction between pumpability and shootability (CCSA-46).

Example 22: The findings of this research ***will*** hopefully guide future efforts to design more thermally stable nanocrystalline alloys (CASA-21).

The use of possibility or prediction modals in examples 21 and 22 indicates the writer's confidence in the findings. *Can* in Example 21 implies that the present research is meaningful and *will* in example 22 suggests the future positive influence of the current research results. Moreover, it is also noteworthy that the use of the verb of effort/ facilitation + *to*-clause in the DTR move of both corpora contains the most frequent occurrence. In examples 23 and 24, the students choose to use verbs of facilitation (*help* and *allow*) to demonstrate their attitude that the results of the present research are valuable, which indicates the importance of the study. For example:

Example 23: These results ***help*** to control the conditions for generating the continuous and uniform carburized layer on the surface and around the pore wall of porous TiAl alloy (CCSA-52).

Example 24: A potential consequence of this study would be the development of a superior armor material that is sufficiently affordable, ***allowing*** it to be incorporated into the general soldier's armor chassis (CASA-33).

Stance adverbs are also common in this move of both corpora, especially likelihood adverbs and style adverbs. For instance:

Example 25: The initial crystalline thickness increase was ***mainly*** due to the crystallization of some tie chains in the amorphous region, as well as small transformation of diffuse transition layer into crystalline region (CASA-17).

Example 26: The remarkably enhanced performance of lithium electrode could ***probably*** be attributed to the effective protection of a unique SEI layer; formed under the synergistic effect of LiFSI and DOL (CCSA-40).

The adverbs in the two examples are used in the interpretation of the research results. The use of style adverbs (*mainly*) in example 25 in this move suggests the writers' attitude that their explanation is rational and acceptable but there may be other possible causes behind the results. And the likelihood adverb *probably* in example 26 is to demonstrate the writers' caution and prudence in the interpretation. Both groups utilize stance adverbs to avoid being assertive in making claims, which demonstrates their respect for opinions from their community and makes their explanation of results more acceptable to the readers.

Chinese and American students have similar preferences in the choice of stance markers in the DTR move of the abstract. On the one hand, they utilize possibility and prediction modals and verbs of facilitation + *to-*clauses to show the positive impacts of the research results. On the other hand, they prefer to use *that* and *to*-clauses controlled by communication verbs and mental verbs as well as style and likelihood adverbs to show their respect for different opinions, which makes the research significance more credible. Both groups employ different stance devices in the DTR move to realize the interpersonal function of the abstract.

## Discussion

Taken together, the findings reveal a similar move structure; but the divergence is apparent in the lexical-grammatical features in the dissertation abstracts by Chinese and American students.

In response to the first research question, most abstracts in both corpora include four or five moves, indicating a comprehensive understanding of the rhetorical features of the writers (Sükan and Mohammadzadeh, 2022). Within those five moves, Presenting the research, Describing the methodology, and Summarizing the findings are found in most abstracts, supporting previous research findings (Santos, 1996; Pho, 2008; Can et al., 2016; Sükan and Mohammadzadeh, 2022). This is especially true for dissertation abstracts in the current study, all of which report empirical studies in the discipline of materials science and engineering in IMRD (introduction-methods-results-discussion) format. In terms of the length of each move, the emphasis on the Summarizing the findings move by both groups conforms with the findings of El-Dakhs (2018), which is a salient feature of scientific discourse. American students put more balanced emphasis on Situating the research, Describing the method, and Summarizing the findings moves in abstracts of journal articles. In contrast, Chinese students focus more on Summarizing the findings. This is different from Ren and Li (2011) finding which shows that Chinese students over-emphasize the introduction move in MA thesis in applied linguistics. The reason for this incongruence can be disciplinary differences. Scientific discourse, as McKenny and Bennett (2011) point out, is characterized by the presentation of facts while social science and humanities rely more on argumentation. The results of the move structure in the abstracts by the two groups suggest a larger effect of disciplinary norms than cultural factors.

In response to the second research question, we first looked at the overall lexical-grammatical features of stance in the dissertation abstracts. No significant differences were found in the total number of stance expressions in CCSA and CASA. Both groups employed stance-complement clauses the most, followed by modal and semi-modal verbs and stance adverbs. This supports Biber (2006) observation of the lexical-grammatical features of the written registers. However, Chinese students use significantly more modals and semi-modals while American students use more stance adverbs. This is in line with Çakir (2016) finding that native writers employ more stance adverbs in abstracts than L2 writers.

Although no significant differences were found between the overall frequencies of stance-complement construction in the CCSA and CASA, the use of stance verb-complement construction differed in the two corpora. Stance verb + *to*-complement construction shares a similar function to *that*-complement construction in abstracts. Both constructions are likely to appear with inanimate subjects to indicate objectiveness, and they are often controlled by verbs with a higher degree of conviction and certainty, such as *find* and *show*. The inanimate subject structure can reveal the authors' confidence in the information, thus collaboratively enhancing the persuasiveness of abstracts. However, compared to their American counterparts, Chinese students tended to limit their choice to *that*-complement construction but underused *to*-complement clauses followed by *find* and *show*. This is in line with Charles (2006) finding that *find/show* verbs are the most frequently used reporting verbs in doctoral theses in material science, displaying the author's detailed knowledge of the research.

In terms of the distribution of stance markers in different moves, the results show that three moves (Situating the research, Summarizing the findings, and Discussing the results) in abstracts in both corpora are heavily loaded with stance markers while the other two moves include few stance features. This supports Pho (2008) finding that rhetorical devices are scarcely found in Presenting the research and Describing the Method moves. Both groups preferred to use possibility modals such as *can* and *may* in Situating the research move to emphasize the importance of their research topics. The employment of modal verbs such as *may* and *can* when summarizing the previous findings to lead to their own study can reduce the definiteness of the claim concerning previous research and take the responsibility off the present writer (Pho, 2008). In Summarizing the findings move, however, possibility modals are used to show the writer's caution about the outcome of the current study. Modals in Discussing the results move have two functions: allowing alternative interpretations of the results (with the use of possibility modals) and emphasizing the significance of the findings (with the prediction modals, such as *will*). This finding is in line with Pho (2008) observation that writers prefer to use modal verbs as hedging or boosting device in the DTR move.

Next to modal verbs, stance verb-complement clauses are the second most preferred stance device employed by both groups, particularly in Summarizing the findings and Discussing the research moves. This is most likely due to the multiple functions of verbs in conveying the epistemic meaning that is dominant in abstracts (Hyland, 2000). It is interesting to note that Chinese students use more stance verb-complement clauses in Discussing the research move while American students use more of them in Summarizing the findings moves. In the Summarizing the findings move, complement clauses followed by *find/show* verbs are used to present the writer's own findings. This is consistent with Pho (2008) finding that the principal syntactic feature of the STF move is the employment of *that*-complement structure controlled by the reporting verbs. In contrast, the complement clauses in Discussing the research move, particularly the verb of effort/ facilitation + *to*-clause, help to highlight the value of the research findings.

Stance adverbs are the least frequent stance markers used by both groups, most of which are found in the Summarizing the findings and Discussing the research moves. The frequent use of stance adverbs (*probably* and *mainly*) in Discussing the research move suggested Chinese and American students' interpretation of the research results in a cautious way. This can be attributed to the tentativeness of researchers when explaining their research findings (Pho, 2008). Despite the shorter length of Discussing the research move, abstracts by Chinese students are more densely loaded with stance expressions, particularly modal verbs, than abstracts by American students. This suggests L2 writers lack confidence when making claims based on the research findings.

Overall, the stance expressions across the five moves used by the two student groups shared similar distributional features. The DTR, STF, and STR moves are more densely loaded with stance expressions than the DTM and PTR moves in both corpora. The different functions of the moves were realized with the use of these stance features, pinpointing the necessity of conducting the study, establishing the credibility of the research findings, or highlighting the significance of the research. This indicates students' good understanding of the rhetorical function of each move and control of the degree of authorial voice expressed in each move. As pointed out by Li (2020), abstracts function to inform and persuade readers. While students in both groups are aware of discipline-specific knowledge of abstracts, the American students seemed to promote their research more consciously with the use of a variety of stance devices than the Chinese students in the PTR, DTM, and STF moves.

## Conclusion

The present study investigated the lexical-grammatical features of stance in English dissertation abstracts written by Chinese and American students majoring in materials science and engineering. Both similarities and differences were identified in the move structure in Chinese and American doctoral students' dissertation abstracts. Abstracts in both CCSA and CASA corpora include four or five moves, but American students' move structure of abstracts is generally more balanced than their Chinese counterparts, with each move present in most abstracts. In contrast, Chinese students' abstracts were characterized by a longer average length with fewer moves. Both groups underscored the significance of DTM (describing the method) and STF (stating the findings) moves in the abstracts. However, compared to their American counterparts, Chinese students concentrated more on STR (situating the research) than PTR (presenting the research) and DTR (discussing the research) in abstracts.

Some similarities and differences in the overall distribution of lexical-grammatical features could be found in the five moves of CCSA and CASA corpora. The distribution of the three main categories of stance expressions was similar in Chinese and American students' abstracts, with stance-complement clauses being the most preferred stance device and stance adverbs being the least used one. Similar features were also found in the distribution of sub-categories of these stance expressions, with both preferring possibility modals, stance verb-complement clauses, and style and likelihood adverbs. However, in comparison with their American counterparts, the Chinese students preferred modal verbs and stance verbs + *that*-clauses to express stance, but they seldom employed stance adverbs and stance verbs + *to*-clauses.

The findings of the present study have pedagogical implications for academic writing. The results suggest that Chinese students face some challenges in the textual organization of English abstracts. Since Chinese students' dissertations in material science are composed in Chinese, the required abstract in English is very likely to be translated from the Chinese abstract, maintaining the textual features of the Chinese version. Therefore, rhetorical moves and stance markers of research article abstracts should be incorporated into the instruction of academic writing (Zhang and Zhang, 2021). Teachers can highlight the importance of these moves in writing an English abstract with both informative and persuasive functions. Discussion on the differences between the writing convention in English and Chinese would benefit L2 students' English abstract writing. Additionally, writing instructors can provide students with feedback on their use of stance markers in academic writing to make stance more visible. Some meaningful findings have emerged from the current study, yet there exist some inevitable limitations. Firstly, the sizes of the corpora are relatively small, which may restrict the generalizability of the findings. Secondly, the study focuses only on a single discipline and may not provide a comprehensive understanding of students' stance features in abstract moves. Therefore, future studies could construct a larger corpus to cover abstracts from different disciplines and conduct cross-disciplinary research on the choice of stance devices in different abstract moves. Moreover, a comparative study on the use of various stance devices in different moves of abstracts by students at different proficiency levels is also expected to provide meaningful findings.

## Data availability statement

The raw data supporting the conclusions of this article will be made available by the authors, without undue reservation.

## Author contributions

YL and JL conceived of the initial idea and designed the study. JL collected the data and analyzed the data. XH drafted the manuscript. YL and XH revised subsequent versions and proofread the manuscript. YL finalized the manuscript for submission as the corresponding author. All authors have read and agreed to the published version of the manuscript.

## Funding

This work was supported by a research grant from China Social Science Research Foundation (Grant No: 19BYY229).

## Conflict of interest

The authors declare that the research was conducted in the absence of any commercial or financial relationships that could be construed as a potential conflict of interest.

## Publisher's note

All claims expressed in this article are solely those of the authors and do not necessarily represent those of their affiliated organizations, or those of the publisher, the editors and the reviewers. Any product that may be evaluated in this article, or claim that may be made by its manufacturer, is not guaranteed or endorsed by the publisher.
